# Investigation on structural, thermal, optical and sensing properties of meta-stable hexagonal MoO_3_ nanocrystals of one dimensional structure

**DOI:** 10.3762/bjnano.2.62

**Published:** 2011-09-14

**Authors:** Angamuthuraj Chithambararaj, Arumugam Chandra Bose

**Affiliations:** 1 Nanomaterials Laboratory, Department of Physics, National Institute of Technology, Tiruchirappalli – 620 015, India

**Keywords:** fiber optic sensor, hexagonal phase, molybdenum oxide, one dimensional rod, phase transition

## Abstract

Hexagonal molybdenum oxide (h-MoO_3_) was synthesized by a solution based chemical precipitation technique. Analysis by X-ray diffraction (XRD) confirmed that the as-synthesized powder had a metastable hexagonal structure. The characteristic vibrational band of Mo–O was identified from Fourier transform infrared spectroscopy (FT-IR). Scanning electron microscopy (SEM) and transmission electron microscopy (TEM) images clearly depicted the morphology and size of h-MoO_3._ The morphology study showed that the product comprises one-dimensional (1D) hexagonal rods. From the electron energy loss spectroscopy (EELS) measurement, the elemental composition was investigated and confirmed from the characteristic peaks of molybdenum and oxygen. Thermogravimetric (TG) analysis on metastable MoO_3_ revealed that the hexagonal phase was stable up to 430 °C and above this temperature complete transformation into a highly stable orthorhombic phase was achieved. The optical band gap energy was estimated from the Kubelka–Munk (K–M) function and was found to be 2.99 eV. Finally, the ethanol vapor-sensing behavior was investigated and the sensing response was found to vary linearly as a function of ethanol concentration in the parts per million (ppm) range.

## Introduction

Considerable research interest has been focused on metastable nanocrystalline materials due to their unusual and enhanced properties as compared to their bulk counterparts. Synthesis of metastable nanocrystals with controlled size and shape has always been one of the most fascinating challenges as the properties of nanomaterials are essentially determined by their phase, shape, size and chemical composition [1]. Recently, molybdenum oxide and its compounds have aroused interest for various potential applications, such as gas sensors, highly reactive catalysts, high optical contrast electrochromic devices and cathodic electrodes for lithium batteries, due to their unique structural, optical and electrical properties [2–5]. Several synthetic techniques have been developed for controlled synthesis of nanocrystalline MoO_3_ materials [6–11]. Recently, Dhage et al. [12], Ramana et al. [13] and Song et al. [14] have prepared h-MoO_3_ and studied its structural and thermal properties. Zheng et al. prepared highly dispersed h-MoO_3_ through a solution-based technique and investigated its photochromic and electrochromic properties [4]. By adjusting the hydrothermal reaction temperature, metastable (h-MoO_3_) and stable (α-MoO_3_) MoO_3_ nanoparticles were successfully synthesized by Chithambararaj et al. [15].

Thus, many research articles are focused on the synthesis of h-MoO_3_ as opposed to the stable α-MoO_3_, due to the reduced crystallite size and one dimensional growth. Usually, the α-MoO_3_ is a stable structure formed at a higher temperature relative to the metastable h-MoO_3_. Thus, there is a phase transformation from h-MoO_3_ to α-MoO_3_ at 400 °C, which is inferred from TGA/DTA studies [14,16]. With an increase in temperature, the crystallites aggregate and promote rapid growth of the particle. Hence, crystallite size in the nanometer range is difficult to control in the case of α-MoO_3_.

The present work demonstrates the synthesis of metastable h-MoO_3_ material through a solution-based chemical precipitation technique. The structure, shape and size of the as-synthesized sample were characterized by XRD, SEM and TEM analysis. The effects of the crystallite size and strain, on the diffraction peak broadening, were deconvoluted and their values were estimated using Scherrer and Wagner–Aqua methods. The phase stability and its transformation into a highly stable orthorhombic structure were confirmed by thermal studies. The optical band structure and ethanol vapor-sensing behavior were studied by means of diffuse reflectance spectroscopy (DRS) and fiber optics spectroscopy, respectively. To the best of our knowledge, this paper reports for the first time the ethanol vapor-sensing mechanism with h-MoO_3_ by fiber optics sensor.

## Results and Discussion

### Crystal phase analysis

**Figure 1:**
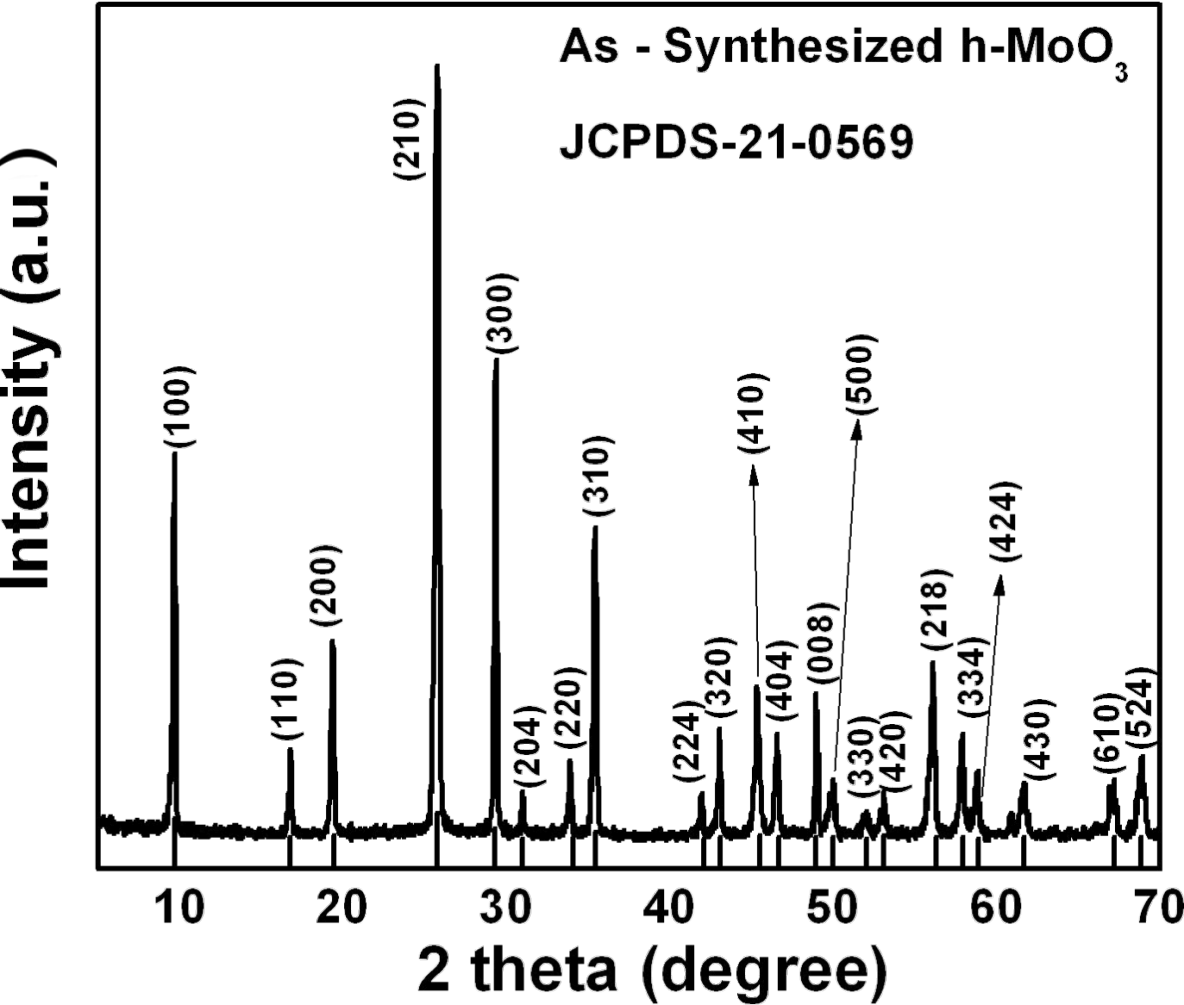
XRD pattern of as-synthesized h-MoO_3_.

The XRD pattern of the as-synthesized MoO_3_ powder is shown in Figure 1. The sample product crystallizes in the hexagonal phase of MoO_3_, and the diffraction peaks are indexed with reference to a standard (JCPDS-21-0569: a = 10.522 Å and c = 14.888 Å) data file. No secondary impurity peaks corresponding to other polymorphs of MoO_3_, namely orthorhombic or monoclinic, are present. From the XRD pattern, the high intensity peak observed at 26.5° is broadened in comparison to the bulk compound. The observed broadening is a convolution of the instrumental error, the reduced crystallite size and the existence of microstrain in the synthesized sample product.

The overall contribution to the broadening is expressed as β_hkl_ = β_ins_ + β_size_ + β_strain_, where β_ins_ is the full width at half maximum (FWHM) due to instrumental error, and β_size_ and β_strain_ are the FWHM due to the crystallite size and strain, respectively. The broadening due to instrumental error is eliminated by the Rachinger method [17]

[1]
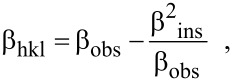


where β_obs_ and β_hkl_ refer to the FWHM of observed and measured sample profiles, respectively. In order to study the crystallite size and strain effect on the peak broadening, Scherrer [18] and Wagner–Aqua (W–A) [17] methods were considered. The Scherrer method is used to calculate the crystallite size (*D*_hkl_) with the assumption of negligible strain,

[2]
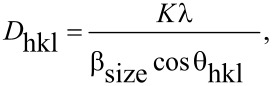


where *K* is the shape factor (0.9), λ is the wavelength of Cu Kα_1_ radiation (1.5406 Å), and θ_hkl_ is the Bragg diffraction angle. The crystallite size was estimated and found to be 51 nm. Although the size of the crystallite is in the nanometer range, significant structural defects such as dislocations, staking faults, twin boundaries and intergrowth, etc., induce strain in the material, which results in considerable broadening of the diffraction peak profile. In order to distinguish and deconvolute the crystallite size and strain effects on the peak broadening, the W–A method is adopted and the equation is

[3]
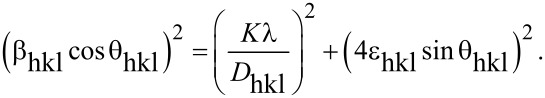


**Figure 2:**
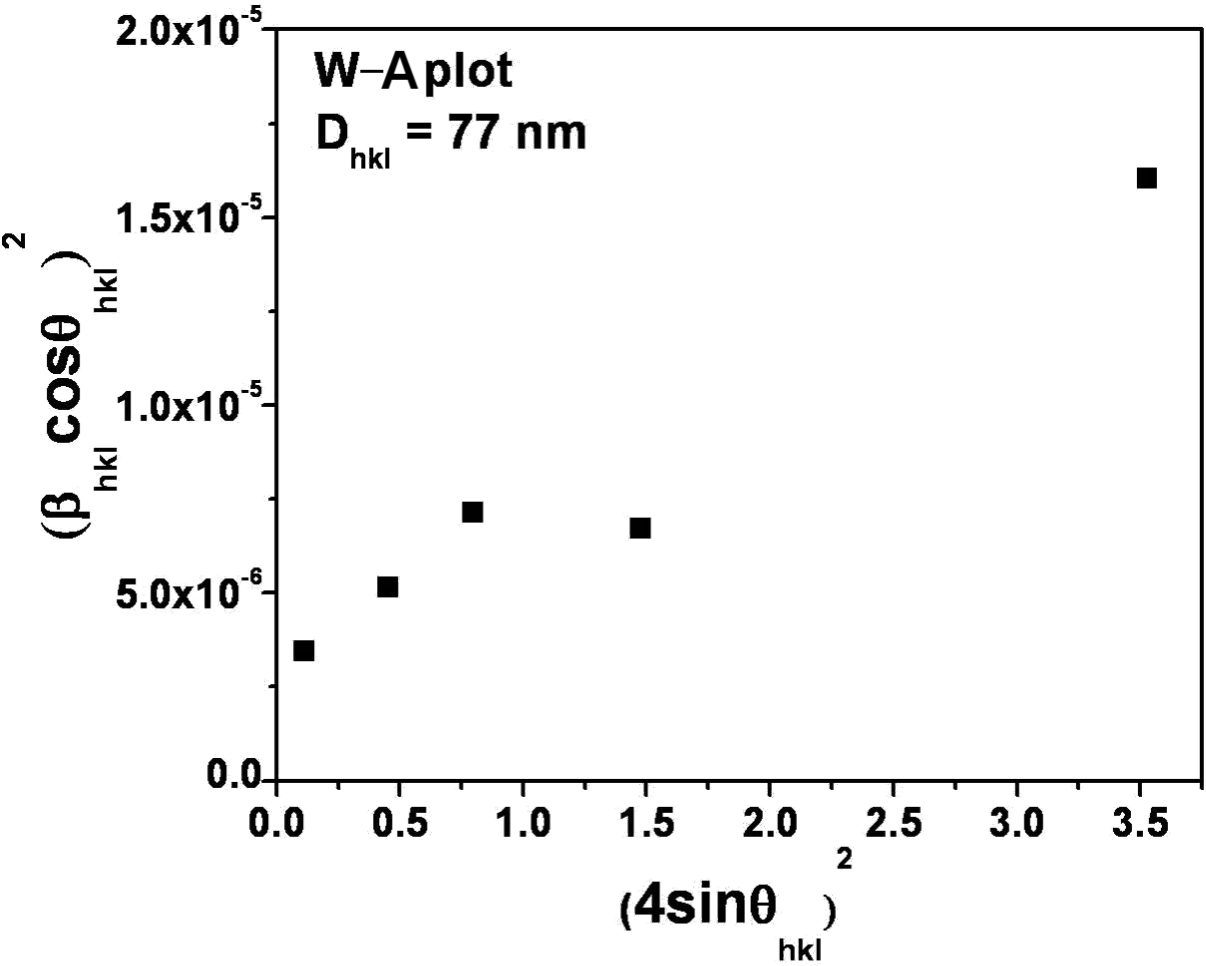
W–A plot of h-MoO_3_ for crystallite size and microstrain estimation.

Figure 2 shows the W–A plot for the as-synthesized sample. To determine the crystallite size and strain, a graph was plotted with (4sinθ_hkl_)^2^ along the X-axis and (β_hkl_cosθ_hkl_)^2^ along the Y-axis. From the linear fit, the crystallite size (*D*_hkl_) was extracted from the Y-intercept and the microstrain (*ε*_hkl_) from the slope of the fit.

The crystallite size and strain were estimated from the graph and the values are 77 nm and 3.509 × 10^−6^, respectively. From these results, it is clearly confirmed that the observed broadening in the XRD peaks is mainly due to the smaller crystallite size.

### Functional group analysis

**Figure 3:**
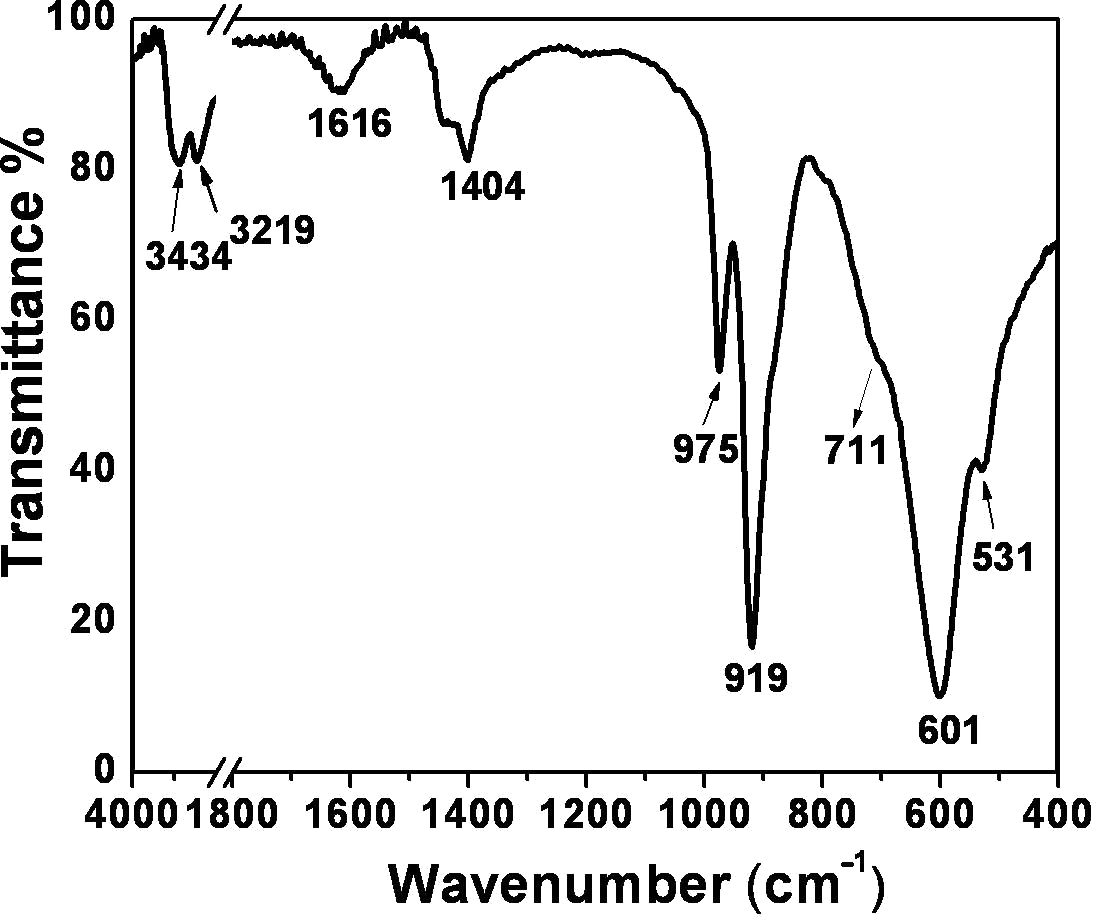
FT-IR spectrum of as-synthesized h-MoO_3_.

The functional groups present in MoO_3_ are identified through FT-IR analysis and are shown in Figure 3. A small band at 3434 cm^−1^ and a sharp band at 1616 cm^−1^ correspond to the stretching and bending vibrations, respectively, of the hydrogen bonded –OH group in water molecules.

Distinct peaks at 3219 cm^−1^ and at 1404 cm^−1^ are attributed to the stretching and bending vibrations of N–H in NH_4_^+^. These results are consistent with the previously reported results [19]. The peaks between 1000 cm^−1^ and 900 cm^−1^ are ascribed to the Mo=O characteristic stretching vibration of the hexagonal phase. A broad and complex band peaked at 600 cm^−1^ corresponds to the Mo–O vibration [14].

### Surface morphology and particle size analysis

**Figure 4:**
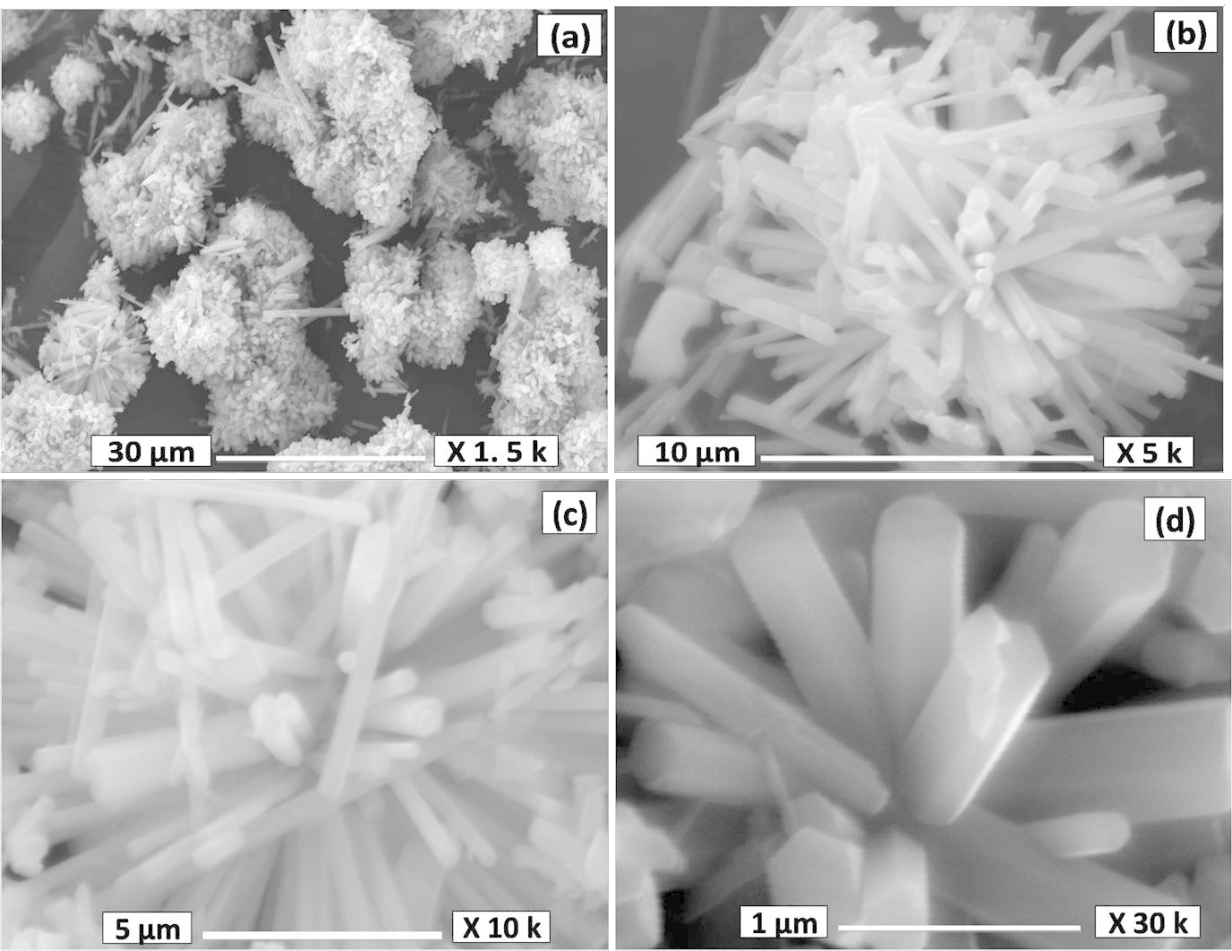
Low and high magnification SEM micrographs of h-MoO_3_.

**Figure 5:**
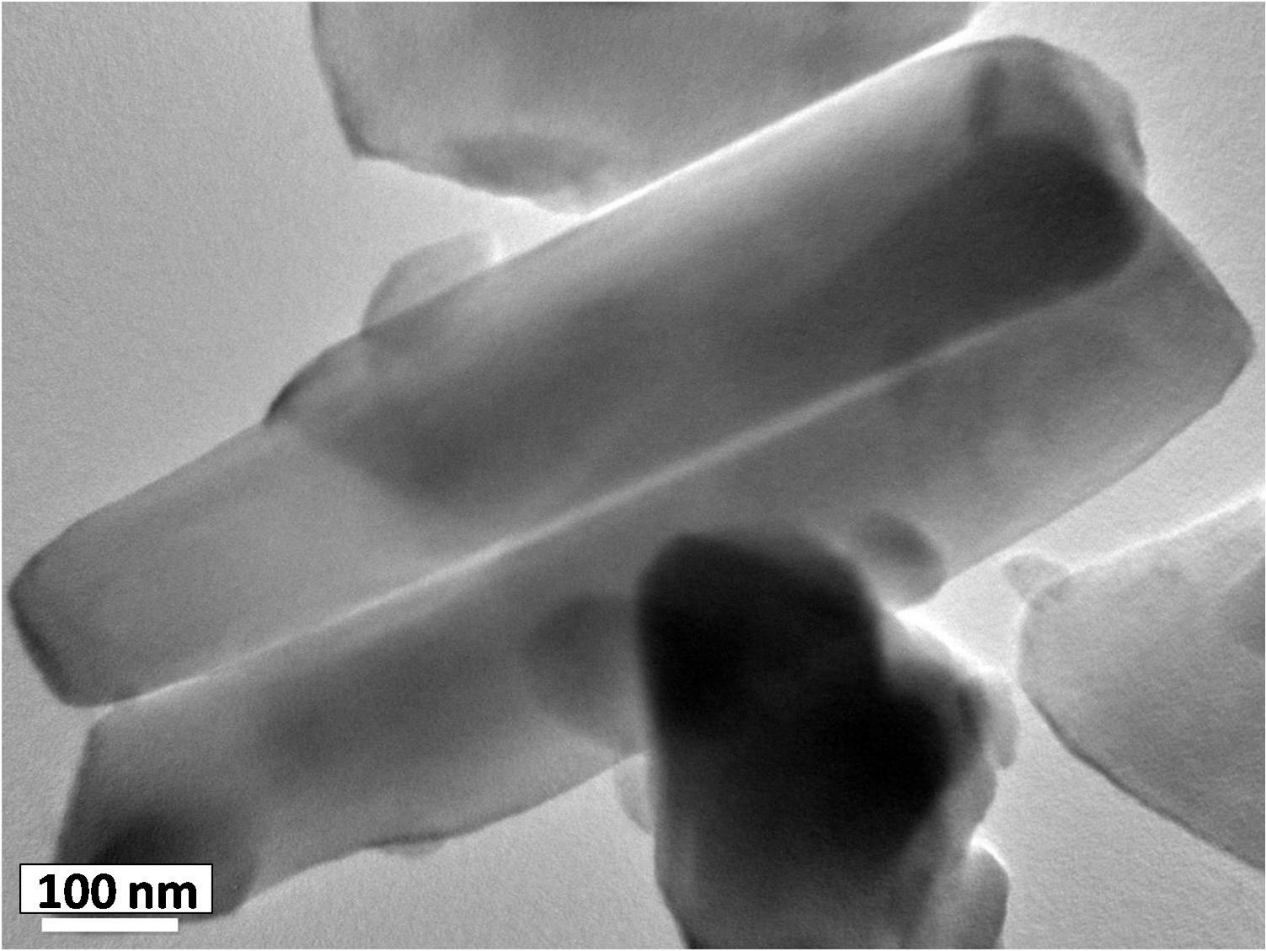
TEM micrograph of the one-dimensional structure of h-MoO_3_.

Figure 4 displays the low and high magnification SEM images of as-synthesized h-MoO_3_ powder. From Figure 4a, the particles are widely distributed and are found to be aggregated in a spherical structure whose diameter is in the micron range and comprises a bunch of nanorods. The corresponding high magnification images (Figure 4b–d) show that the particles are in the form of one-dimensional hexagonal rods. A typical TEM image of the as-synthesized particle is shown in Figure 5. It clearly illustrates that the as-synthesized powder comprises 1D structures of ~200 nm diameter and length of about 800 nm.

**Figure 6:**
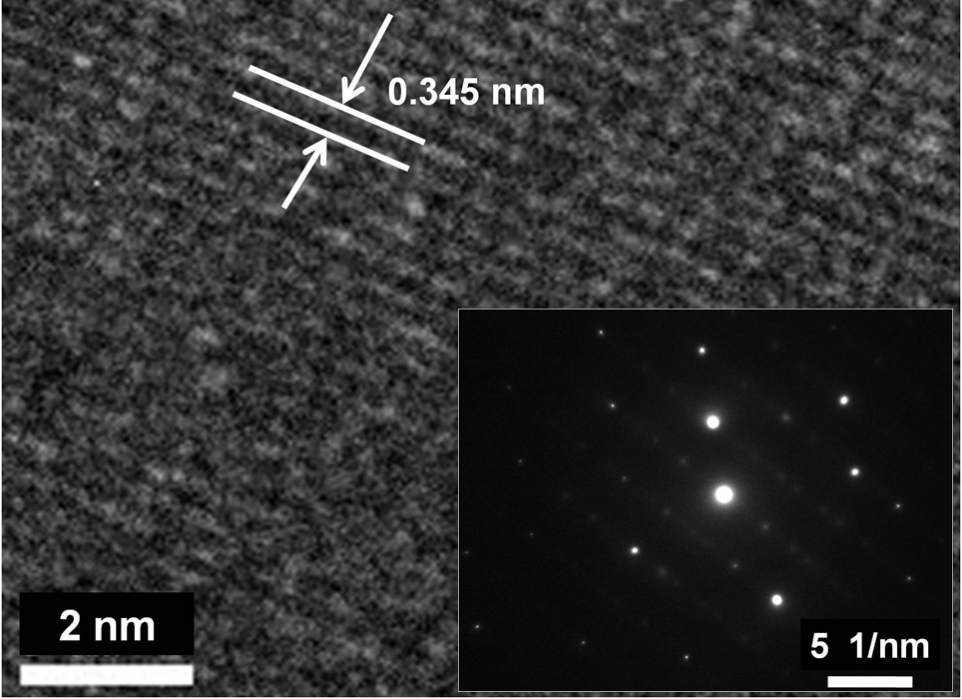
HRTEM image of an as-synthesized h-MoO_3_ nanorod and their electron diffraction pattern (inset).

Figure 6 shows the HRTEM image and the inset is the corresponding SEAD pattern of h-MoO_3_. From the lattice resolved HRTEM image, the distance between the two planes was found to be 0.345 nm, belonging to the (210) plane of h-MoO_3_. The electron diffraction, with a highly intense dotted pattern, reveals the single crystalline nature of h-MoO_3_. Furthermore, the elemental composition and chemical bonding information were confirmed by EELS investigation.

**Figure 7:**
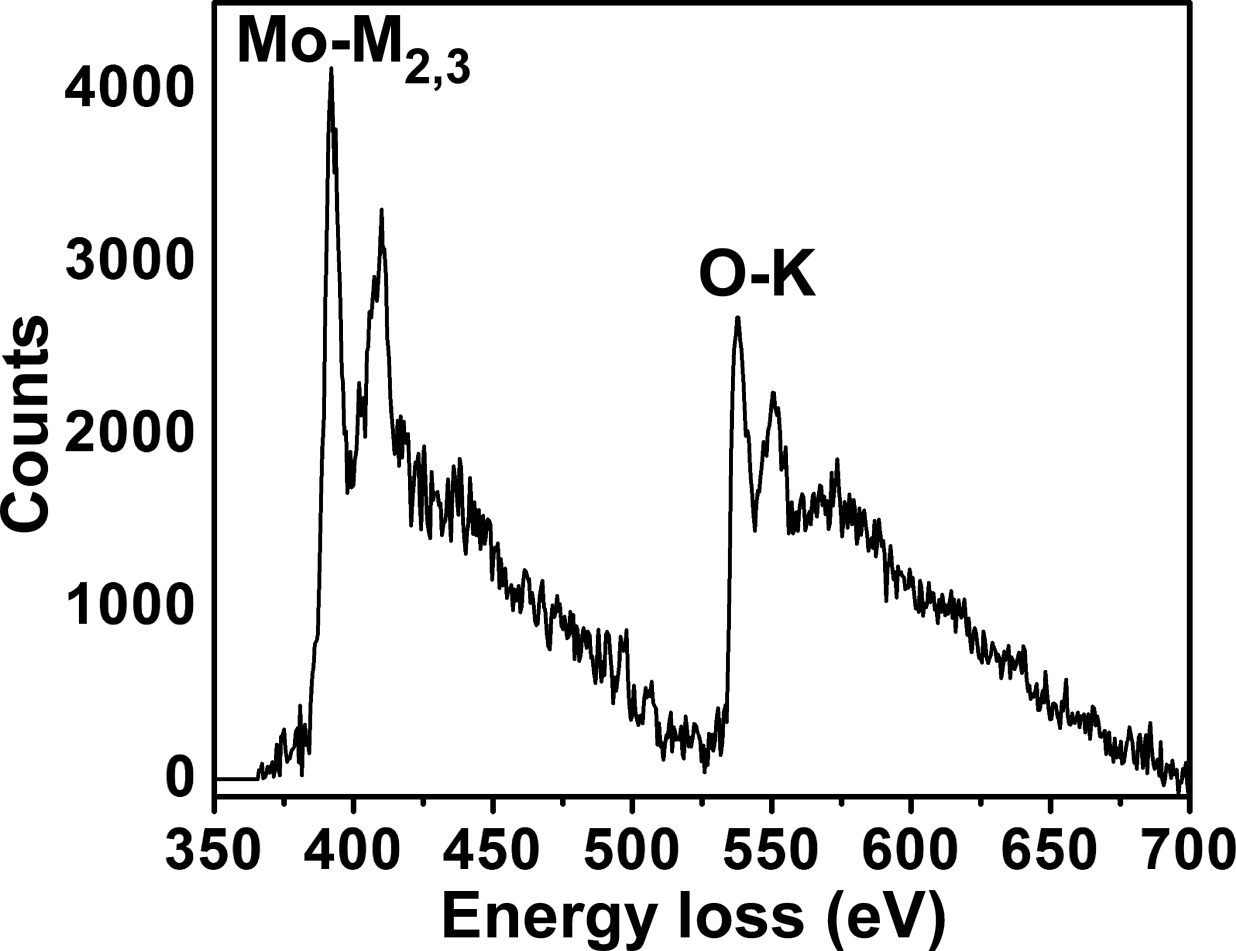
EELS spectrum of resultant h-MoO_3_.

Figure 7 depicts the typical EELS profile of an individual hexagonal rod. It shows two distinct edges at 532 eV (oxygen K edge) and 382 eV (molybdenum M edge), confirming the presence of only O and Mo with a covalent interaction [20]. The observed O-K edge peak reflects the electronic transitions from the oxygen 1 s core level to the unoccupied final state of O 2p hybridized with the Mo 4d state [21], and the Mo-M_2,3_ peak is due to excitation from 3p_1/2_ and 3p_3/2_ core level electrons to unoccupied states (4d and 5s) of molybdenum [22].

### Growth mechanism and formation of 1D h-MoO_3_

On the basis of experimental results, the possible mechanism for the formation of h-MoO_3_ is outlined as follows: In the present work, (NH_4_)_6_Mo_7_O_24_·4 H_2_O and HNO_3_ are used as reactant precursors for the synthesis of h-MoO_3_ nanocrystals. As per the following reaction Equation, MoO_6_ is considered as an initial seed nucleus for the formation of MoO_3_.

(NH_4_)_6_Mo_7_O_24_·4 H_2_O (aq) + 6 HNO_3_ → 7 MoO_3_ (s) + 6 NH_4_NO_3_ (aq) + 7 H_2_O

Here, molybdenum is located at the center and six oxygen atoms are coordinated in octahedral sites. During the nucleation, these octahedral structures interact with each other through corners (a-axis) and edges (c-axis) by means of electrostatic interactions between NH_4_^+^ and OH^−^ ions. The stacking and assembling of these octahedral structures result in the formation of a stable hexagonal structure. Thus, the NH_4_^+^ functional group present in the reaction condition acts indirectly as a structure directing agent [4,23]. Moreover, the initial seed nuclei formed during the nucleation stage are composed of hexagonal unit cells which can induce anisotropic growth along the c-axis, and thus hexagonal shaped nanocrystals with 1D structure are favored [24–25].

### Thermal analysis

**Figure 8:**
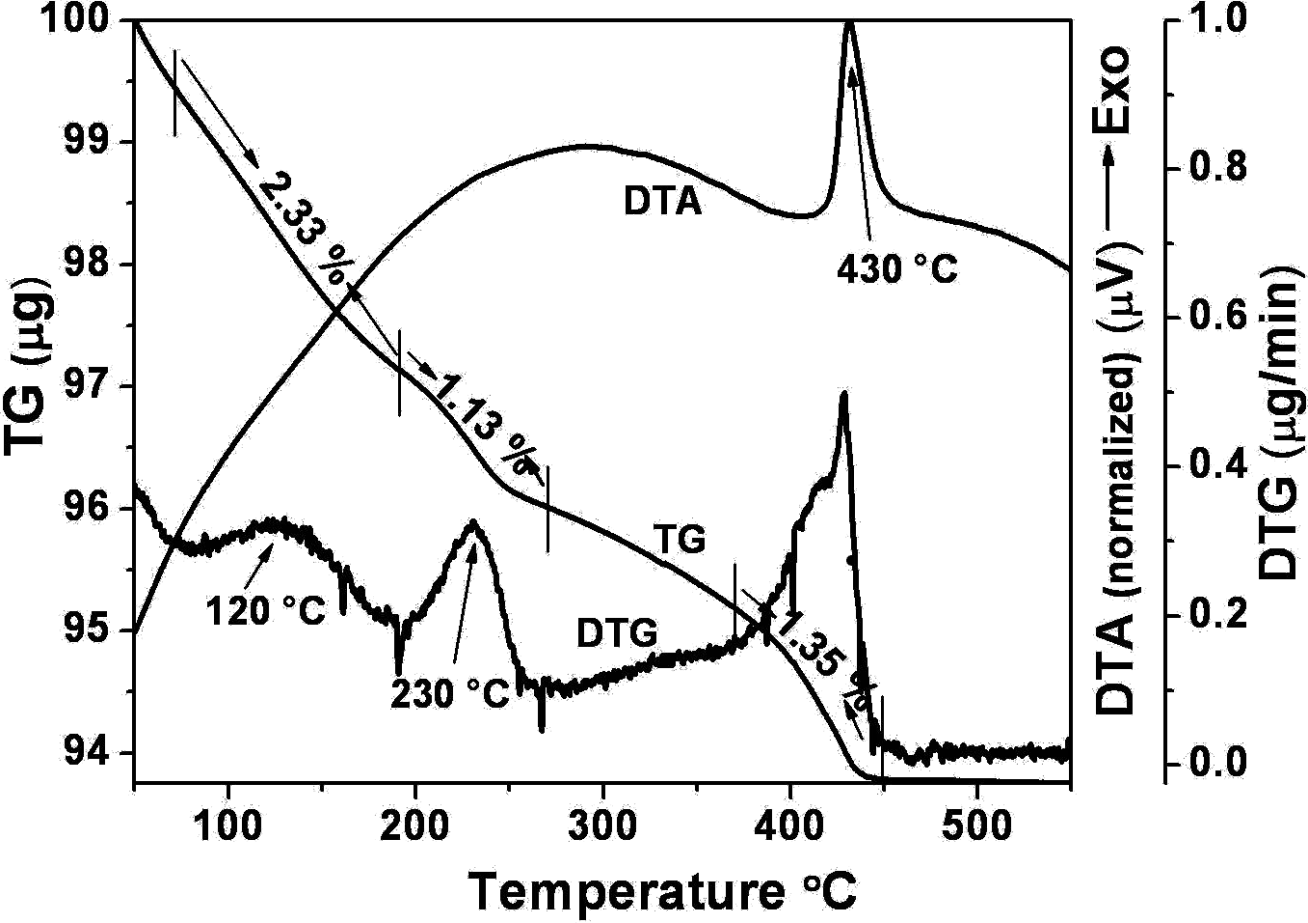
TG/DTG and DTA curve of h-MoO_3_.

The thermal behavior was studied in detail and is depicted in Figure 8. From the TG and DTG graph, the first weight loss (2.33 wt %) in the 70–190 °C region corresponds to vaporization of the hydrogen bonded water molecules that are physically adsorbed on the surface of the MoO_3_. The second weight loss (1.13 wt %) takes place between 190 and 270 °C, due to vaporization of gaseous nitrates and ammonia compounds from the material.

The same observation is made from the DTA curve, which exhibits two broad exothermic peaks well below 270 °C. After dehydration and removal of ammonia compounds, a sharp exothermic peak at 430 °C with weight loss of 1.35 wt % is attributed to liberation of coordinated water and ammonia molecules from the internal structure of the MoO_3_ material, which promotes an irreversible phase transformation from the hexagonal to the orthorhombic structure [14,16]. The powder subjected to TGA measurements was subjected to XRD analysis, and the result confirmed that above 450 °C the product is in a stable orthorhombic structure (Figure not included). Above 450 °C no changes in the TG/DTA curve are seen revealing that the thermodynamically stable α-MoO_3_ was achieved.

### Optical absorption studies

**Figure 9:**
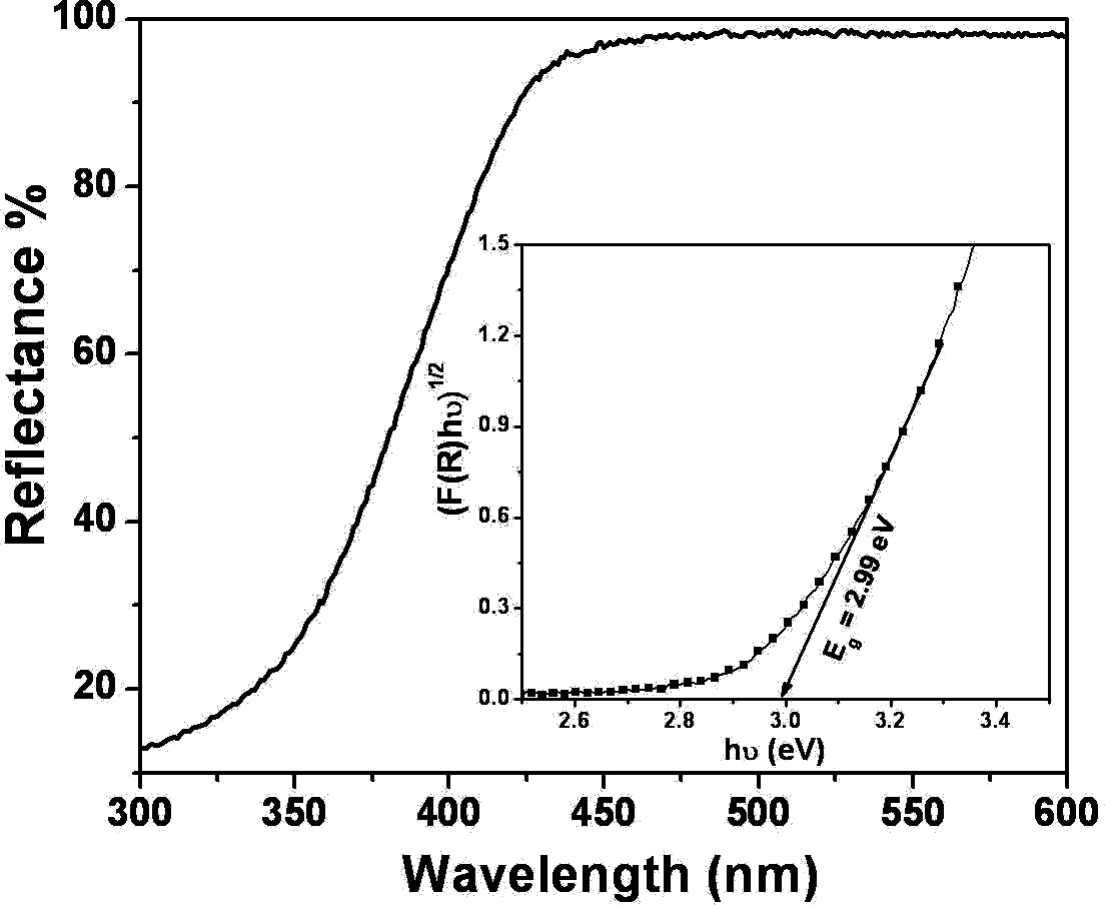
DRS spectrum of h-MoO_3_ (Inset: optical band gap energy of h-MoO_3_).

The optical absorption behavior and band gap energy of h-MoO_3_ was studied by means of DRS, as shown in Figure 9. The spectrum shows a maximum reflectance in the region between 600 nm and 450 nm corresponding to lower absorption. At 420 nm, a decrease in reflectance is seen due to fundamental absorption (valance band to conduction band) by the material [26]. Further, the optical band gap is evaluated using K–M function as follows [27]:

[4]
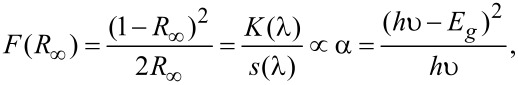


where *F*(*R_∞_*) is the K–M function or re-emission function, *R_∞_* is the diffuse reflectance of an infinitely thick sample, *K*(λ) is the absorption coefficient, s(λ) is the scattering coefficient, *hν* is the photon energy and *E*_g_ is the band gap energy for indirect transition.

The indirect optical band gap energy is determined by extrapolating the linear portion of the plots of (*F*(*R_∞_*)*hν*)^1/2^ versus (*hν*) shown as an inset in Figure 9. The estimated energy band gap value is about 2.99 eV and is considerably higher than that of the bulk (2.95 eV). This increase in *E*_g_ is attributed to the reduced particle size.

### Sensor study

**Figure 10:**
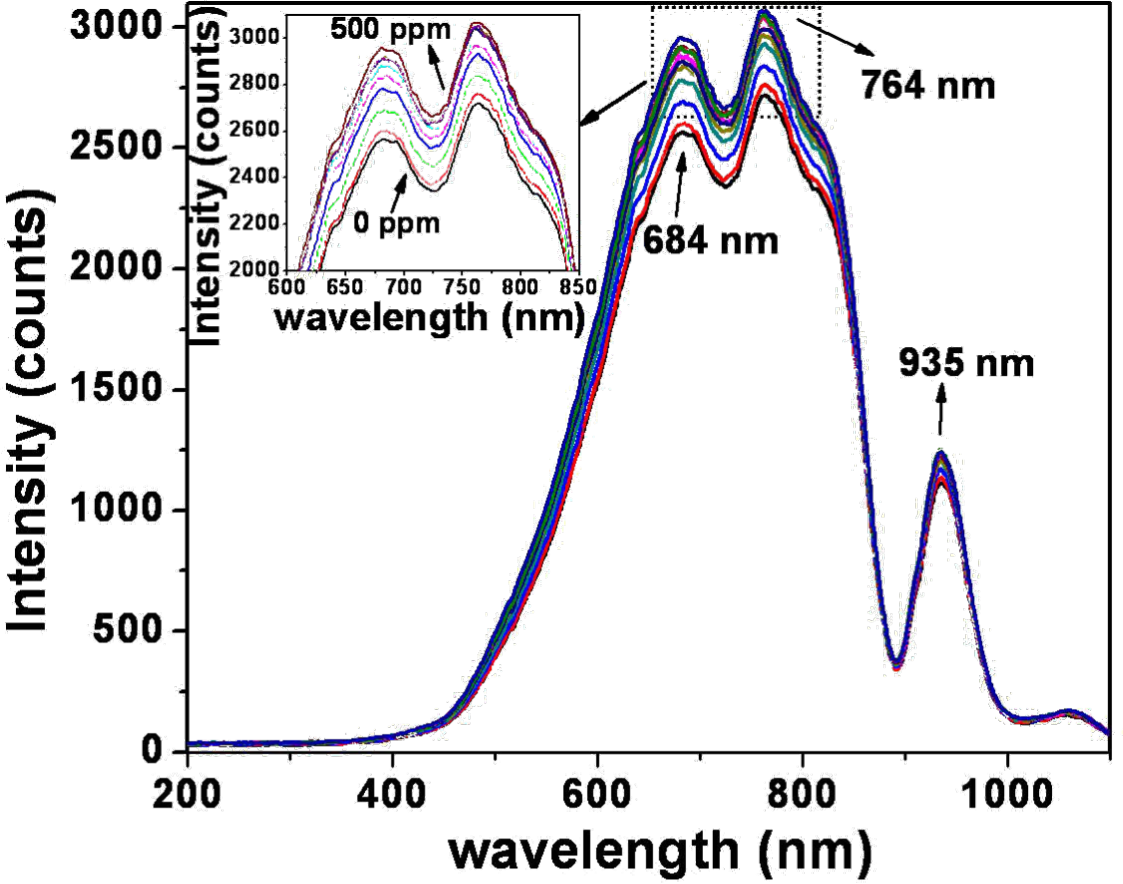
Spectral response of h-MoO_3_ for varying concentration of ethanol in ppm.

The ethanol vapor-sensing behavior was examined by means of an optical fiber sensor setup and analyzed on the basis of simultaneous changes in the refractive index and evanescent wave absorption. In our experiment, a portion of the fiber cladding (~3 cm) was partially removed and coated with as-synthesized h-MoO_3_; this is considered as the sensing area. In the sensor study, the characteristic spectrum as a function of wavelength in the range of 200–1100 nm was recorded for different ethanol concentrations. The recorded spectrum is shown in Figure 10 and the inset represents the magnified view of the spectra recorded in the range between 600 and 800 nm. In this plot, the Y-axis denotes the intensity, measured in counts, for each concentration of ethanol. The ethanol concentration was increased from 0 to 500 ppm in increments of 50 ppm. From Figure 10, it is clearly seen that the intensity peaks are a maximum at 684, 764 and 935 nm. The magnified spectrum, shown as an inset, clearly shows the gradual increase and variation in intensity with increase in ethanol concentration. The gas sensitivity is calculated using the formula

[5]
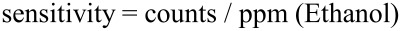


**Figure 11:**
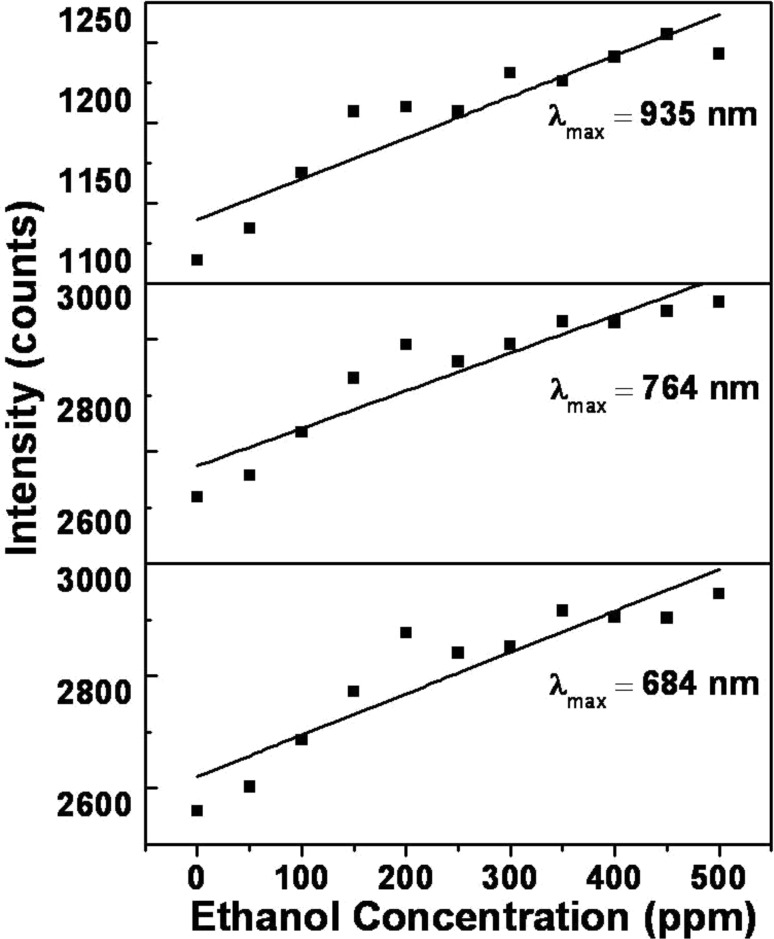
Plot of peak intensity versus ethanol concentration for values λ_max_ = 684, 764 and 935 nm.

The sensitivity is estimated by plotting a separate graph of ethanol concentration (in ppm) versus intensity (in counts) for each value of λ_max_ (684, 764 and 935 nm respectively). The plot is depicted in Figure 11: The slope of the curve determines the sensitivity and is found to be 74, 67 and 26 counts/ppm for λ_max_ = 684, 764 and 935 nm, respectively. From the plot, the maximum sensitivity is seen for λ_max_ = 684 nm.

Here, it is proposed that the gas sensing mechanism follows the changes in the refractive index and evanescent wave absorption. For h-MoO_3_, the oxygen vacancies and interstitial molybdenum atoms play a major role in gas sensing operation. The reaction between ethanol gas and the chemisorbed oxygen (O_2_^−^, O^−^ and O^2−^) at the surface of the MoO_3_ material changes the refractive index of the clad structure. Due to this change, the light wave travelling through the removed and coated portion of the cladding experiences a change in the evanescent wave absorption [28–32]. Thus, the resultant intensity recorded in the spectrum is a sum of changes due to refractive index and evanescent wave absorptions.

## Conclusions

In summary, metastable h-MoO_3_ was prepared by a solution-based chemical precipitation technique. The metastable hexagonal phase was confirmed by XRD. The crystallite size of the h-MoO_3_ was in nanometer range and the corresponding strain was in the order of 10^−6^. The metal–oxygen vibrational states (M=O and M–O) were identified from the vibrational spectrum. The one-dimensional hexagonal rod structure, with regular arrangement and preferred orientation, was observed in SEM, TEM and HRTEM images. From EELS, the O-K edge and Mo-M_2,3_ peaks confirmed the existence of molybdenum and oxygen. From thermal analysis, the structural transformation from hexagonal to stable orthorhombic was observed at 430 °C. A formation and growth mechanism for the h-MoO_3_ nanocrystals with 1D structure was proposed. The estimated band gap was 2.99 eV from the optical absorption studies. Finally, the ethanol vapor-sensing behavior was investigated and the sensor response increased linearly as a function of ethanol concentration in the range of 0 to 500 ppm. In the present study, changes in the refractive index and the evanescent wave absorption phenomenon were proposed to explain the gas sensing mechanism. The reaction between ethanol gas and the chemisorbed oxygen (O_2_^−^, O^−^ and O^2−^) at the surface of the MoO_3_ material simultaneously changes the refractive index and evanescent wave absorption thereby varying the intensity at the detector.

## Experimental

All the chemicals were purchased from Merck and were used without any further purification. Ammonium heptamolybdate tetrahydrate (AHM) was dissolved in distilled water (10 mL), to give a concentration of 0.2 M. A homogeneous solution was obtained after stirring for 15 minutes and was then mixed with concentric nitric acid (5 mL). The mixture was then heated at 85 °C for 1 h and the resulting precipitate was subsequently washed and centrifuged with distilled water several times. The obtained powder was dried at 70 °C in vacuum for 6 h. Thus, by a chemical precipitation technique, the h-MoO_3_ nanocrystal was synthesized by acid decomposition of ammonium molybdate according to the following reaction equation,

(NH_4_)_6_Mo_7_O_24_·4 H_2_O (aq) + 6 HNO_3_ → 7 MoO_3_ (s) + 6 NH_4_NO_3_ (aq) + 7 H_2_O.

The crystalline phase was analyzed by XRD using a Ultima III Rigaku X-ray diffractometer with Cu Kα_1_ radiation (1.5406 Å), in the range of 5–80° in steps of 0.2 °/min. We eliminated the peak broadening due to instrumental error by performing control experiments with a standard silicon sample and hence accounting for the error properly in the estimation of the crystallite size and strain. FT-IR was recorded on a PerkinElmer spectrometer. For FT-IR measurements, the pellet was prepared by mixing synthesized powder with KBr, and the spectra were recorded in the range of 4000–400 cm^−1^. To examine the surface morphology and particle shape, the powder was uniformly sprayed on carbon tape; a gold coating was made on the sample for 30 s and the micrographs were obtained at various magnifications by means of SEM (Model S3000-Hitachi). TEM, high resolution TEM (HRTEM) images and EELS spectrum were acquired by means of a JEOL model JEM FX II 2000 instrument. The thermal behavior was analyzed by means of an EXSTAR6200 thermal analyzer at a heating rate of 10 °C/min, from room temperature to 550 °C in air. The weight loss and the corresponding phase transition were observed by thermal gravimetric (TG), differential thermal gravimetric (DTG) and differential thermal analysis (DTA). The optical properties of h-MoO_3_ were studied by DRS and were recorded by means of a computer controlled T90+ UV–vis spectrophotometer. A BaSO_4_ plate was used as a reference (100% reflectance), on which a fine-ground powder was pressed. The spectrum was recorded at room temperature in the wavelength range 230–700 nm at a rate of 1 nm/s. Ethanol vapor sensing was performed with a fiber optics sensor arrangement with a white light source (Model SL1, StellarNet Inc., USA with wavelength range from 200 to 2000 nm) and a miniature fiber optics spectrometer (EPP-2000, StellarNet Inc, USA) having a spectral range of 100 to 1100 nm. Poly methyl methacrylate (PMMA) based multimode plastic step index fiber (length 100 cm, diameter 700 µm and numerical aperture 0.51) with cleaved ends was used. The clad region of the fiber was partially removed (about a length of 3 cm) and the surface was polished. The polished surface was coated with h-MoO_3_. A round bottom flask (1000 mL) was used as the gas chamber into which the fiber optic sensor was inserted. An ethanol solution of concentration 0–500 ppm was prepared and used in the gas chamber for sensor measurement.
